# Definition of critical skin defect and concepts of structural and functional repairs: Proposal and verification in a rat model

**DOI:** 10.1002/ame2.70075

**Published:** 2025-09-14

**Authors:** Cong Sun, Weihong Guo, Fang Liang, Rabia Javed, Weijian Hou, Xingdong Zhang, Qiang Ao

**Affiliations:** ^1^ Department of Tissue Engineering, School of Intelligent Medicine China Medical University Shenyang Liaoning China; ^2^ Department of Nursing Tieling Health Vocational Collage Tieling Liaoning China; ^3^ Xuanwu Hospital Capital Medical University Beijing China; ^4^ NMPA Key Laboratory for Quality Research and Control of Tissue Regenerative Biomaterial, Institute of Regulatory Science for Medical Device, National Engineering Research Center for Biomaterials Sichuan University Chengdu Sichuan China

**Keywords:** critical defect, functional repair, quantitative evaluation, skin defect, structural repair

## Abstract

**Background:**

Rats are often used to prepare skin defect models. However, the skin defect sizes of the models prepared by researchers are different, and the lack of consensus on the critical‐size defect makes it difficult to compare their research results.

**Methods:**

The time for wound closure was evaluated and recorded through gross observation. The regression equation between the healing time and the diameter of skin defect was established, which can be used to predict the healing time for a certain skin defect size in rats. Histochemical and immunohistochemical staining was used to observe the regeneration and reconstruction of skin appendages, and the functional skin repair was quantitatively scored.

**Results:**

The critical‐size defect of rats was determined based on the maximum capacity of structural skin repair, and the functional skin repair was quantitatively scored based on the regeneration and reconstruction of skin appendages. The allowable range of critical‐size skin defect of SD rats lies between 45 and 50 mm in diameter. The concept of structural repair and the category of functional repair of injured skin are put forward. The regression equation between the structural skin healing time and defect diameters is established.

**Conclusion:**

The allowable range of skin critical‐size defect of SD rats lies between 45 and 50 mm in diameter. The regression equation between the structural skin healing time and defect diameters can be used to predict the healing time for a certain skin defect size in rats.

## Graphical abstract

This graphical abstract illustrates the research process regarding skin defect repair using a rat model of skin defect. First, it assesses whether the barrier function of the skin is repaired. If the answer is “YES”, the study proceeds to focus on structural repair. During this stage, the concept of structural repair is established, and a regression equation of skin healing time and defect area is formulated. Subsequently, the concept of functional repair is explored, and a quantitative score of skin functional repair is determined. Conversely, if the barrier function is not repaired “NO”, the situation is classified as “Not healing”, which leads to the definition of critical skin defect. This flow ‐ chart comprehensively presents the key steps and concepts in the study of skin defect repair, integrating both structural and functional aspects of the repair process.

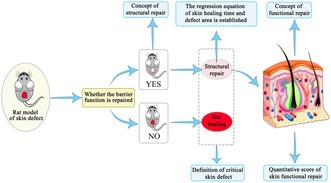

## 1 INTRODUCTION

Skin is the largest organ of the human body, accounting for about 7% of the total body weight. It is composed of epidermis, dermis, and other appendages derived from epidermal cells, such as nails, hair, and glands.^1^ Skin is an anatomical barrier between the external environment and the internal organ system of the human body. It plays a vital role in maintaining the balance of the body and protecting the internal organs.^2^, ^3^

Since ancient times, skin damage has perplexed human beings due to its complexity.^4^, ^5^ Different species have different self‐repair abilities after skin injury. Animals such as zebrafish, tadpoles, and salamanders have perfect skin regeneration ability,^6^, ^7^, ^8^ but in mammals, the perfect skin regeneration (complete recovery of normal skin structure) only occurs in early embryos.^9^, ^10^, ^11^, ^12^, ^13^ After skin injury in adult mammals, the repair is usually completed by fibrous hyperplasia, which leads to the formation of scar.^14^, ^15^, ^16^ Animal and human fetal trauma models show that gestational age is not the only factor affecting skin regeneration and repair, but the size of the wound also plays an important role in wound repair.^17^, ^18^ If the skin defect area exceeds a critical value, even scar healing cannot be achieved,^19^, ^20^, ^21^ and the size of this critical defect area is named critical defect of the skin.

Exploring various new treatment measures and their clinical efficacy for skin injury needs scientific methods and systematic verification.^22^ Therefore, it is necessary to select and prepare appropriate animal models for the evaluation of treatment strategies before they are put into clinical use. Small animals such as rodents,^23^, ^24^, ^25^ rabbits,^26^, ^27^, ^28^ pigs,^29^, ^30^, ^31^, ^32^ dogs^33^, ^34^, ^35^ are often used as in vivo models to evaluate skin wound healing.^36^ In the study of skin wounds, the dorsum of rats is a common site for preparing skin wound models. Although rat's skin is different from humans in anatomical structure and healing ability, it has been used as the first choice of an animal model to verify the effect of wound treatment because of its high genetic similarity with humans, convenient feeding, and low cost.^37^, ^38^, ^39^, ^40^, ^41^, ^42^ In the previous studies of rat dorsum skin defect models, the size and shape of the wounds were different. For example, the diameter of circular defect area varies from 6 mm to 3 cm,^43^, ^44^, ^45^ and the square area varies from 1 to 4 cm^2^.^46^, ^47^, ^48^ In addition, the applied rat strains and ages are different,^33^, ^49^, ^50^ and the rat skin critical defect is not clearly defined. Therefore, it is important to define the critical defect value of the skin and establish a standardized animal model of critical‐size skin defect to ensure the consistency of the evaluation system.

Different sizes of skin defects have different healing outcomes.^51^ If the defect area exceeds the critical size, it will not heal. Within the range of critical defects, the skin can achieve structural closure, including scar healing, which is defined as skin structural repair. Based on structural repair, if there is some regeneration of skin appendages, it involves the reconstruction of complex skin functions, which belongs to the category of functional repair. In addition, there is a lack of systematic research on the evaluation of skin functional repair.

Therefore, in the present study, rat skin defect models were prepared to explore the characteristics of skin healing under different sizes of skin defects. The critical‐size defect of rats was determined based on the maximum capacity of skin structural repair, and the skin functional repair was quantitatively scored based on the regeneration and reconstruction of skin appendages.

## 2 METHODS

### 2.1 Animals and experiment groups

The study was approved by the Animal Experiment Ethics Committee of China Medical University (CMU2019251) (provided in Appendix S1).

### 2.2 Histopathological and morphometric analysis

**FIGURE 1:**
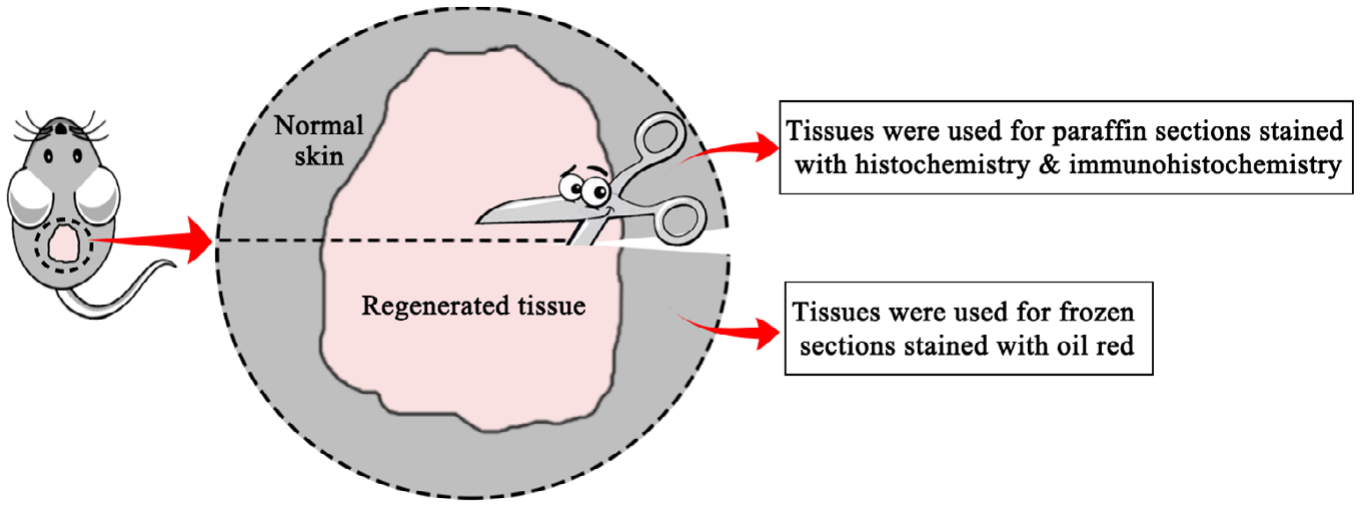
Schematic illustration for specimen harvest.

Histopathological and morphometric analysis (Figure 1) (provided in Appendix S1).

### 2.3 Immunohistochemical evaluation

**FIGURE 2:**
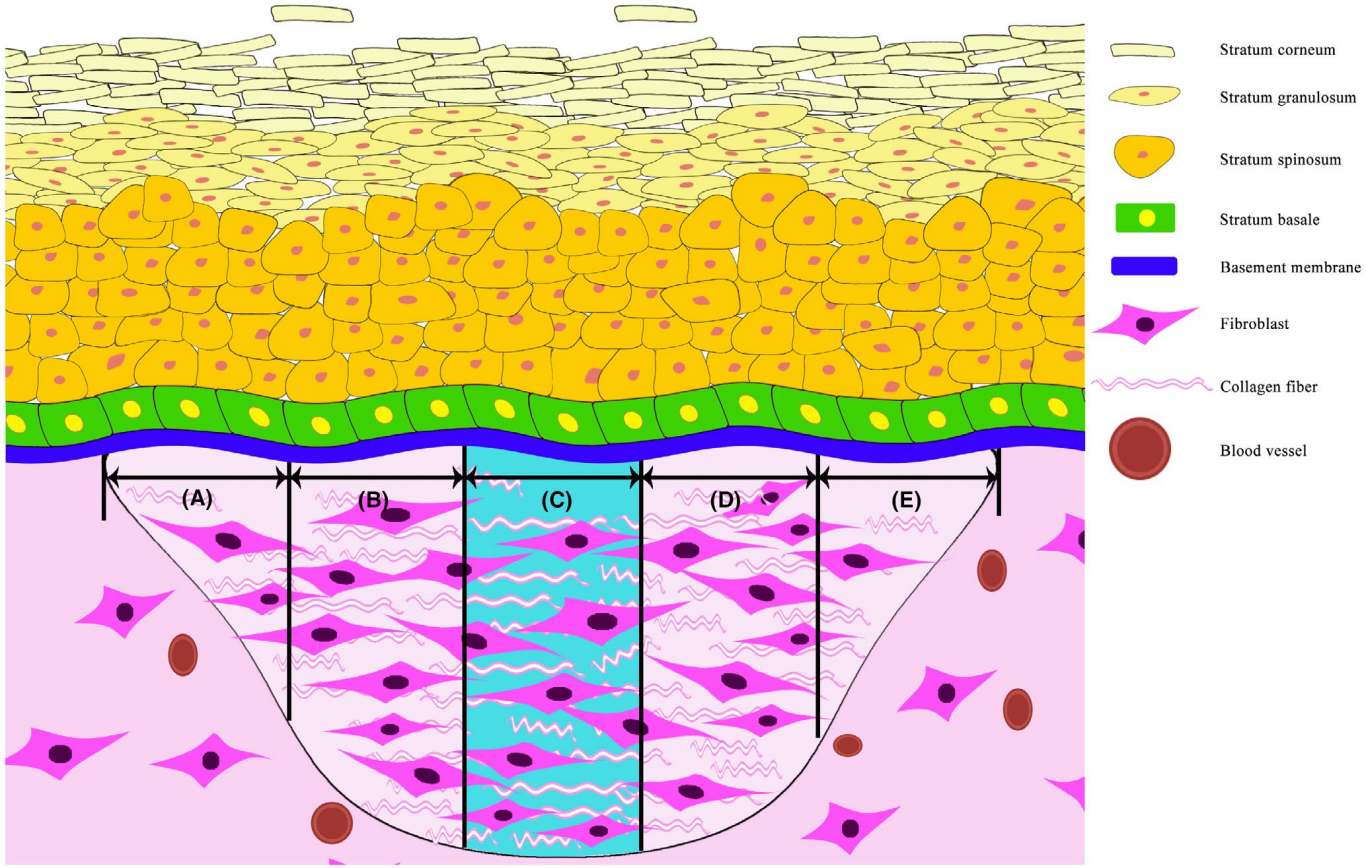
Schematic diagram of skin structural repair. (A, B, C, D, and E) represent five equal parts of the width of regenerated tissue.

The regenerated tissue was divided into five equal parts: A, B, C, D, and E (Figure 2). The DAB positive expression in each part was observed and photographed for skin functional repair score (provided in Appendix S1).

### 2.4 Assessment for skin functional repair

The functional skin repair was quantitatively scored (Table 1). Epidermis thickness is an indicator of skin pathological changes. The regenerated epidermis usually thickens with varying degrees. The higher the degree of thickening, the worse the quality of healing. The basement membrane, hair follicle, and sebaceous gland are important accessory structures of normal skin. Their regeneration and reconstruction represent the recovery degree of skin function. As shown in Figure 2, the regenerated tissue is divided into five areas: A, B, C, D, and E. The results of chemical staining and immunohistochemical staining were observed in each area to quantitatively score the regenerated epidermis thickness and the reconstruction of the basement membrane, hair follicles, and sebaceous glands (Table 1). The higher the score, the better the effect of skin healing, and the score for normal skin is 16.

**TABLE 1:** Scores of skin functional repair.

Score	Epidermis	Basement membrane	Hair follicles	Sebaceous glands
4	Normal	Normal	Normal	Normal
3	Mild thickening	Exists in C	Exists in C	Exists in C
2	Moderate thickening	Exists in B/D	Exists in B/D	Exists in B/D
1	Severe thickening	Exists in A/E	Exists in A/E	Exists in A/E
0	No structural repair	None	None	None

*Note*: Scores in the areas A, B, C, D, and E in Figure 2, and the highest index score for each specimen are recorded. The higher the score is, the better the skin repairs, and the total score for normal skin is 16.

### 2.5 Statistical analysis

All methods for data and fitting analysis of the change of structural repair time with the skin defect diameter are described in the Methods section (provided in Appendix S1).

## 3 RESULTS

### 3.1 Gross observation

The rats with defect diameters ≤45 mm achieve structural closure of the wound. In the group with a defect diameter of 50 mm, nine rats died at days 7–14, only one survived till 110 days after operation, but without skin structural closure (Figure S1A).

When the skin defect diameters are greater than 8 mm, the general appearance of scar shows a greater shrinkage rate in the left and right direction than in the head and tail direction (Figure S1B).

### 3.2 Fitting results of skin structural repair time and defect diameters

#### 3.2.1 Establishment and screening of regression models between skin structural repair time and defect diameters

**FIGURE 3:**
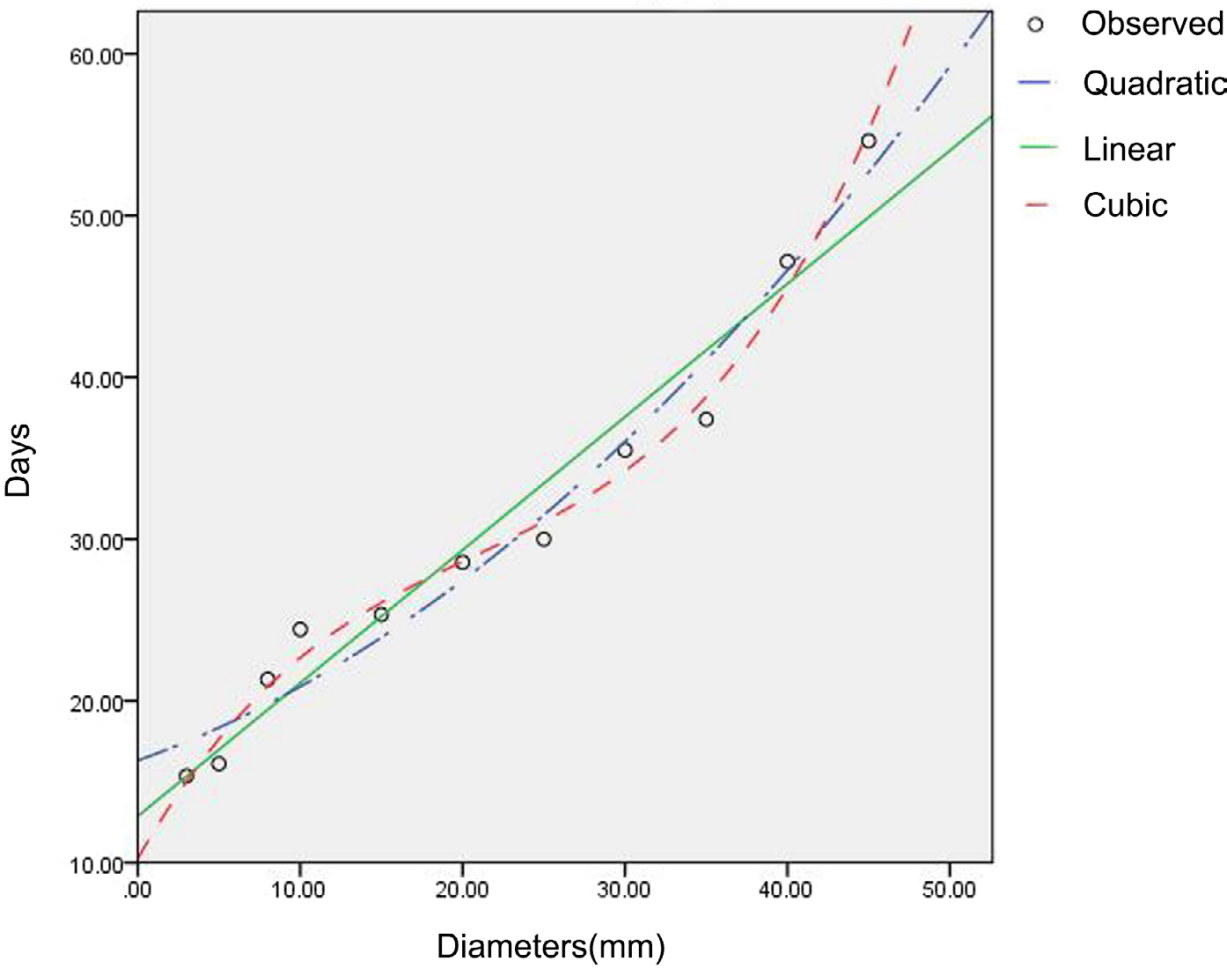
Three fitting curves of the regression equation for skin structural repair time and defect diameters.

Figure 3 shows the scatter diagram of the average days required for skin structural repair with the changes in defect diameters. Figure 3 roughly shows a curve trend. Therefore, curve estimation is used to obtain 11 regression models (Table S1).

From the above 11 regression models, as per judgment, coefficient *R* ^2^ reaches above 0.92, and the significance test of *F* value is less than 0.001. Cubic, quadratic, and linear models are selected (Figure 3).

The selection indicators of the optimal regression model include Root Mean Square Error (RMSE), accuracy factor (A_f_), and bias factor (B_f_). The calculation results of the above three indicators are provided in Table 2. A_f_ and B_f_ in the three models are close to 1, and RMSE in the cubic model is the smallest, indicating that the cubic model is the best.

**TABLE 2:** Results of RMSE, A_f_, and B_f_ in three regression models.

Models	RMSE	Accuracy factor (A_f_)	Bias factor (B_f_)
Linear	0.047	1.003	1.115
Quadratic	0.051	1.013	1.123
Cubic	0.041	0.981	1.099

#### 3.2.2 Establishment of the optimal model equation for the relationship between skin structural repair time and defect diameters

Table 3 shows the parameter estimates of the cubic model. The equation of the cubic model is *y* = 9.835 + 1.818*x* − 0.065*x* ^2^ + 0.001*x* ^3^ (3 mm ≤ *x* ≤ 45 mm).

**TABLE 3:** Parameter estimates of cubic model.

Variables	Unstandardized coefficients		Standardized coefficients	*t*	Significance
	B	SE	Beta		
*x*	1.818	0.253	2.134	7.177	0.000
*x* ^2^	−0.065	0.013	−3.529	−5.114	0.000
*x* ^3^	0.001	0.000	2.450	5.862	0.000
Constant	9.835	1.267		7.761	0.000

#### 3.2.3 Validation of the optimal regression model equation of the relationship between skin structural repair time and defect diameters

Based on the above cubic model equation, the predicted time of structural repair with different defect diameters is calculated, and then the relative error and absolute error between the predicted time of the cubic model and the observed skin structural repair time are calculated (Table 4).

**TABLE 4:** Predicted time results and errors of the cubic model.

Defect (mm)	Observed structural repair time (day)	Predicted structural repair time (day)	Relative error (%)	Absolute error
3	15.00 ± 1.60	14.73	0.32	0.27
5	16.12 ± 1.55	17.42	7.66	1.31
8	21.33 ± 2.00	20.73	3.74	0.60
10	24.00 ± 1.63	22.51	5.79	1.49
15	25.33 ± 1.63	25.85	2.05	0.53
20	28.57 ± 3.30	28.19	0.27	0.38
25	30.00 ± 2.23	30.28	1.28	0.29
30	35.50 ± 2.07	32.87	7.12	2.63
35	37.40 ± 1.14	36.71	1.75	0.69
40	47.16 ± 4.53	42.55	9.11	4.61
45	54.60 ± 3.28	51.14	4.86	3.46

The relative error is 0.27%–9.11%, and the absolute error is 0.27–4.61 days. There is a difference between the predicted value and the observed value, as the skin structural repair time of rats is not only related to its own skin defect diameters but also affected by individual differences and other factors.

**FIGURE 4:**
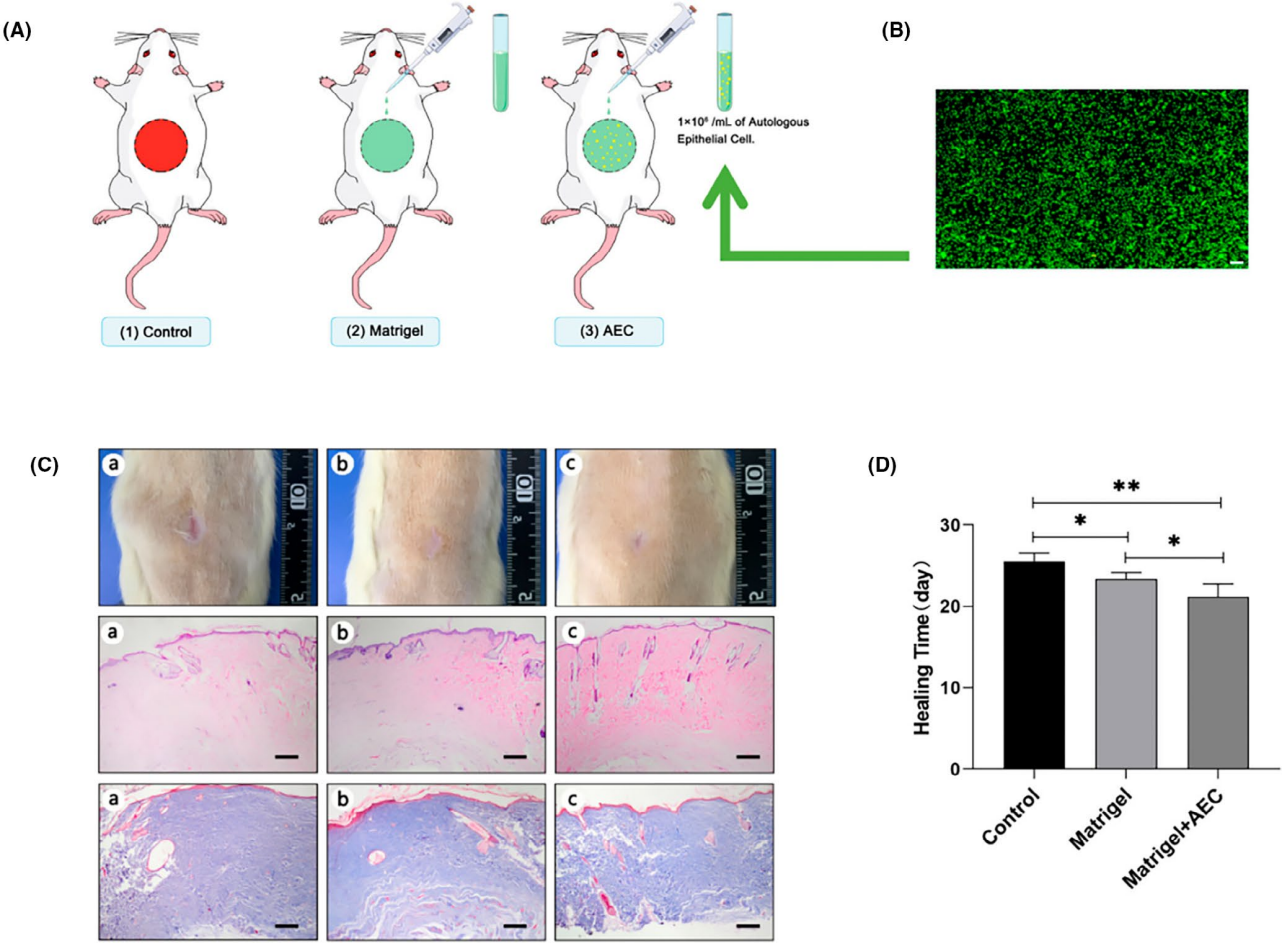
The results of three groups of animal models. (A) Control group (spontaneous healing); matrigel group (400‐μL matrigel covers defect); AEC group (autologous epithelial cells + matrigel). An amount of 200‐μL of matrigel is inoculated with 200‐μL cell suspension with cell density of 2 × 10^6^/mL, and each rat is injected on the wound bed with 400 μL into the wound with cell density of 1 × 10^6^/mL. (B) Representative pictures of autologous epithelial cell apoptosis during AO/PI staining assay. The images were captured at 40× magnification. The lower right scale bar is 200 μm. (C) Representative images of gross and pathological observation of the three groups. (a) represents the control group, (b) represents the matrigel group, and (c) represents the AEC group. The images were captured at 40× magnification. The lower right scale bar is 200 μm. (D) The results of wound healing time of the three groups (*p < 0.05; **p < 0.01).

To verify the rationality and feasibility of the optimal regression model, we added three groups of animal models with skin defect diameter of 13 mm, which was not included in the above fitting sample diameters. Then the time required for skin structural repair was recorded, and the relative error and absolute error were calculated using the optimal regression equation. The three groups of animal models are as follows: (1) control group (spontaneous healing), (2) matrigel group (matrigel covers defect), and (3) AEC group (autologous epithelial cells + matrigel) (Figure 4A). The results of AO/PI staining show that the primary rat epidermal cells proposed by two‐step enzymatic method have high activity (Figure 4B). The structural closure of the wound is achieved in the three groups by gross and pathological observation (Figure 4C). Results after treatment (Figure 4D) show the increased wound healing time. The AEC group showed 21.16 ± 1.60 days; the better matrigel group showed 23.33 ± 0.81 days; and the difference between the two groups has statistical significance (*p* < 0.05). In particular, the healing time of AEC group is significantly shortened than the control group's 25.50 ± 1.04 days, with a significant difference (*p* < 0.01). The predicted days for skin structural repair with defect diameter of 13 mm are 24.68 days using the equation of cubic model, and the average days for skin structural repair are 25.50 ± 1.04 days. The relative error and the absolute error between the predicted skin structural repair days and the observed skin structural repair days are 0.82% and 3.07%, respectively, and within the range of relative error (0.27%–9.11%) and absolute error (0.27–4.61), which proves that the application of this optimal cubic model to predict the time for skin structural repair with different defect diameters is reasonable and feasible.

### 3.3 Histological observation

#### 3.3.1 Hematoxylin and eosin staining

**FIGURE 5:**
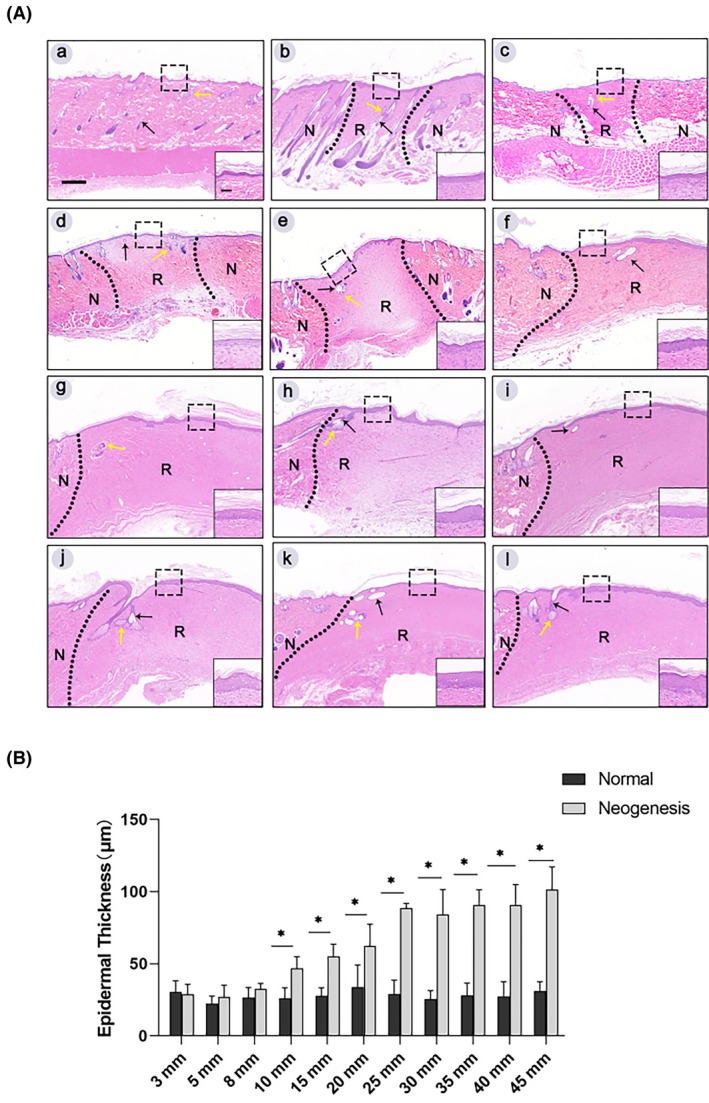
(A) Representative images of hematoxylin and eosin (H&E) staining. In the image, (a) represents normal skin, and (b–l) represent the groups with defect diameters of 3–45 mm, respectively. The black dotted line is the boundary between the right regenerated tissue and left normal tissue. N refers to normal tissue. R refers to regenerated tissue. Black arrow points to the hair follicle. The yellow arrow points to the sebaceous gland. The images were captured at 40× magnification. The lower left scale bar is 200 μm. The high magnification image in the lower right corner comes from the black dotted box. The images were captured at 400× magnification. The scale bar in the high magnification image of the lower left corner is 20 μm. (B) The results of thickness analysis of normal epidermis and regenerated epidermis (*p < 0.001).

As shown in Figure 5A, we observe that normal skin includes the epidermis, epidermal accessory structures, organs, and dermis. A single hair follicle or hair follicle connected to a sebaceous gland can be seen in the dermis. The wounds in each group have been epithelized, and the epidermis has been regenerated. At the site of the new tissues, there are slender collagen fiber bundles arranged in waves parallel to the horizontal of the epidermis, and a large number of cells are dispersed within the collagen fiber bundles in parallel. Two weeks after the closure of the skin structure, the morphological structure of accessory organs, such as hair follicles and sebaceous glands, existed in 20% of the central area of the regenerated tissue in the groups with skin defect diameters of less than 20 mm, but not in the groups of 20 mm or more than 20 mm.

The thickness comparison of the regenerated epidermis of different groups is shown in Figure 5B. The regenerated epidermis thickness is larger than normal. The normal epidermis is composed of three to four layers of cells, namely, basal layer, spinous cell layer, granular layer, and stratum corneum. The cells in the basal layer are columnar and closely arranged. The regenerated epidermis has more of two to three cell layers than the normal one, and the morphology of epidermal cells is uneven. The quantitative analysis of epidermis thickness shows that the regenerated epidermis thickness in groups of skin defect diameters ≤8 mm is slightly higher than the normal epidermis (*p* > 0.05). In the groups of skin defect diameters ≥10 mm, the thickness of the regenerated epidermis is significantly higher than the normal (*p* < 0.05).

#### 3.3.2 Oil Red O

**FIGURE 6:**
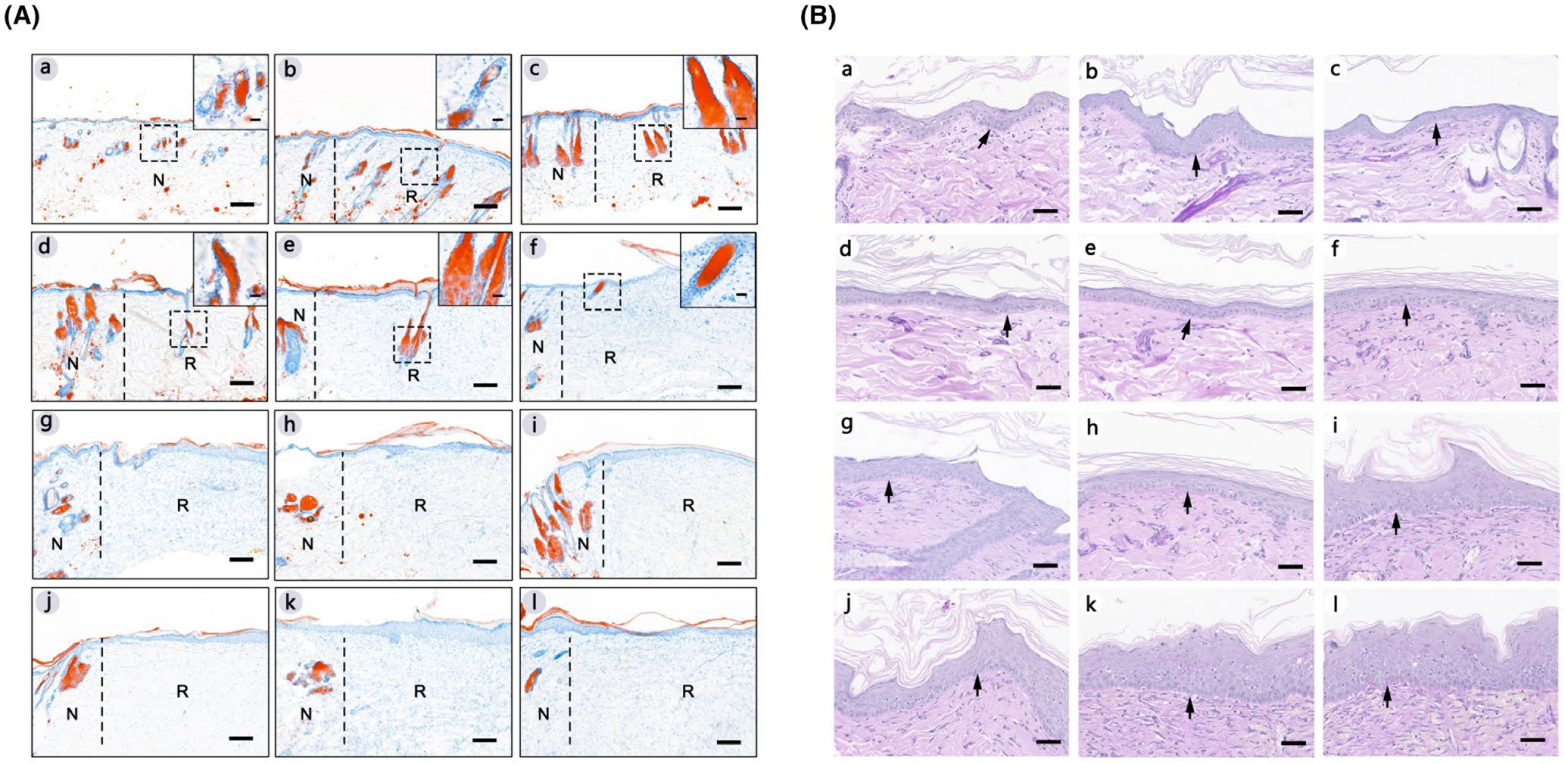
(A) Representative images of sebaceous glands Oil Red O (ORO) staining. (a) represents normal skin and (b–l) represent the groups with defect diameters of 3–45 mm, respectively. On the left of the black dotted line is normal skin, and on the right is regenerated tissue. N refers to normal tissue. R refers to regenerated tissue. The images were captured at 40× magnification. The scale bar in the lower right corner is 200 μm. The high magnification image in the upper right corner comes from the black dotted box. The images were captured at 400× magnification. The scale bar in the high magnification image of the upper right corner is 20 μm. (B) Representative images of periodic acid‐Schiff (PAS) staining. (a) represents normal skin, and (b–l) represent the groups with defect diameters of 3–45 mm, respectively. Black arrow points to the basement membrane. The images were captured at 40× magnification. The Scale bar in the right corner is 200 μm.

The sebaceous glands of the healed dermal tissues in each group are stained with Oil Red O. Figure 6A shows that there is a sebaceous gland staining positive structure in 20% of the central area of the regenerated tissue in the group with skin defect diameters of 3–15 mm, and there is no obvious positive staining in 20% of the central area of the regenerated tissue in the group with skin defect diameters of 20–45 mm, which is similar to the results of H&E staining.

#### 3.3.3 Periodic acid‐Schiff

The regeneration of the basement membrane is present in repaired skin (Figure 6B). In normal skin, periodic acid‐Schiff (PAS) staining of basement membrane shows the continuous purplish‐red line between the epidermal layer and the dermis. In the regenerated skin tissue, the basement membrane is discontinuous.

#### 3.3.4 Masson

**FIGURE 7:**
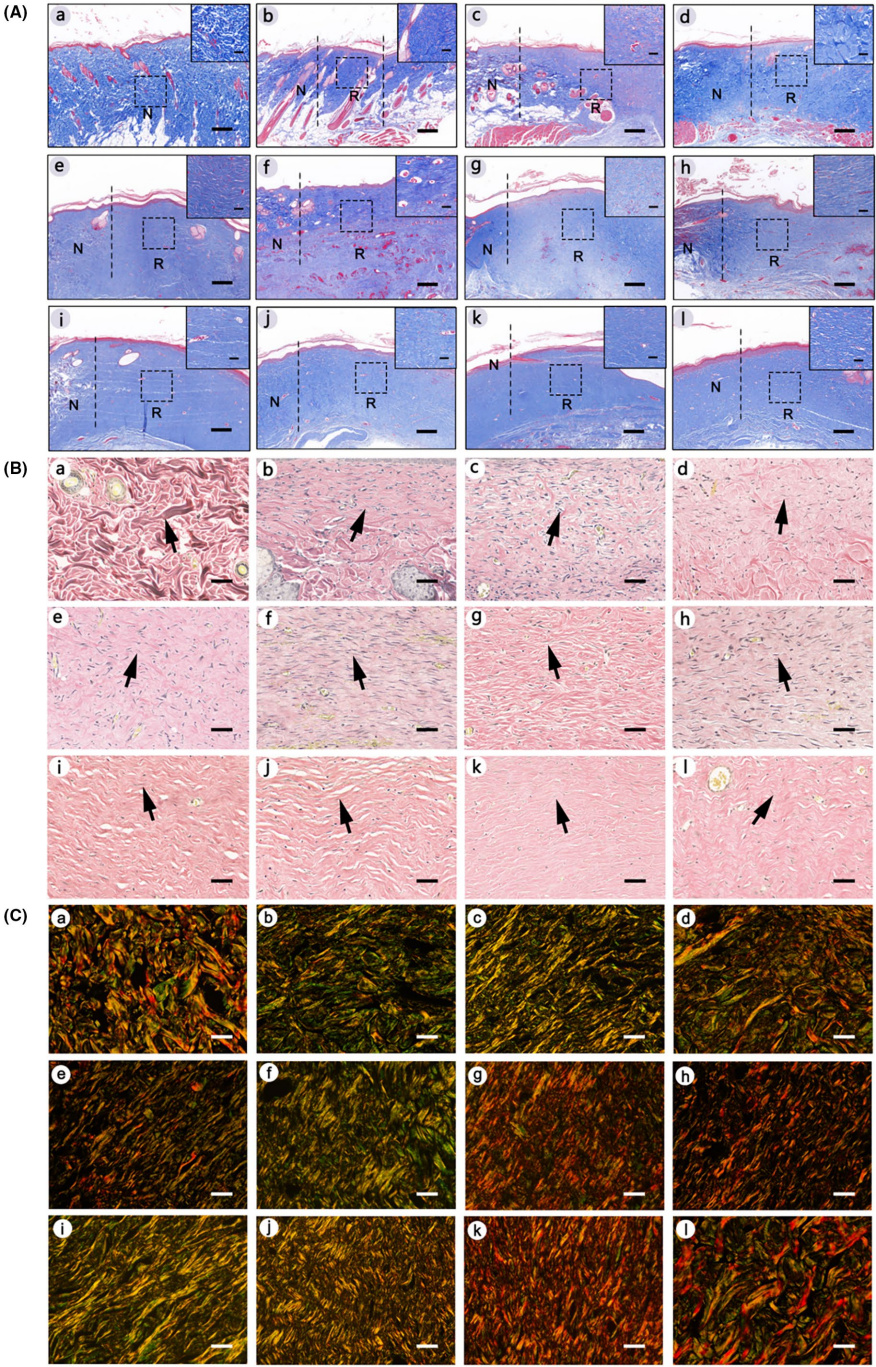
(A) Representative images of Masson staining. (a) Represents normal skin, and (b–l) represent the groups with defect diameters of 3–45 mm, respectively. Red refers to keratin, muscle fiber, or cytoplasm, and blue refers to collagen. On the left of the black dotted line is normal skin, and on the right is regenerated tissue. N refers to normal tissue. R refers to regenerated tissue. The images were captured at 40× magnification. Scale bar in the right corner is 200 μm. The high magnification image in the upper right corner comes from the black dotted box. The images were captured at 400× magnification. The scale bar in the high magnification image of the upper right corner is 20 μm. (B) Representative images of Elastic Verhoeff‐Van Gieson (EVG) staining. (a) Represents normal skin, and (b–l) represent the groups with defect diameters of 3–45 mm, respectively. Black refers to elastic fiber, red refers to collagen fiber, and other components refer yellow. The yellow arrow points to elastic fiber. The images were captured at 400× magnification. The scale bar in the lower right corner is 20 μm. (C) Representative images of PSP staining. (a) Represents normal skin, and (b–l) represent the groups with defect diameters of 3–45 mm, respectively. The long strip orange/red refers to type I collagen fiber, and the loose filament blue/green refers to type III collagen. The images were captured at 400× magnification. The scale bar in the lower right corner is 20 μm.

Masson staining (Figure 7A) of the healing tissue shows the arrangement and deposition of collagen. Histological observation shows that collagen in the dermis of normal rats is rich and orderly (Figure 7A‐a). In the group with defect diameters of 3–10 mm, the arrangement of collagen is loose, and the collagen is thicker and arranged more regularly, which is similar to normal skin tissue. In the group with defect diameters of 15–45 mm, the arrangement of collagen is dense, and the arrangement direction is roughly parallel to the epidermal direction, or the arrangement is relatively disordered.

#### 3.3.5 Elastic Verhoeff‐Van Gieson

The regeneration of elastic fiber is present in repaired skin (Figure 7B). In normal skin, elastic fiber staining (Figure 7B‐a) shows nuclei, collagen, and long elastic fibers. With the increase in the skin defect diameters, the number of elastic fibers gradually decreases, the coloring gradually weakens, and the fibers appears thin, twisted, and disorderly arranged.

#### 3.3.6 Picrosirius‐polarization

The regeneration of type I and type III collagen in regenerated tissues is evaluated by picrosirius polarization (PSP). Figure 7C shows that both type I and type III collagens have been regenerated in each group. Type I collagen and type III collagen are intertwined into a network. Type I collagen is thicker and closely arranged, surrounded by a small amount of green thinner type III collagen, which is loosely arranged.

### 3.4 Immunohistochemical analyses

**FIGURE 8:**
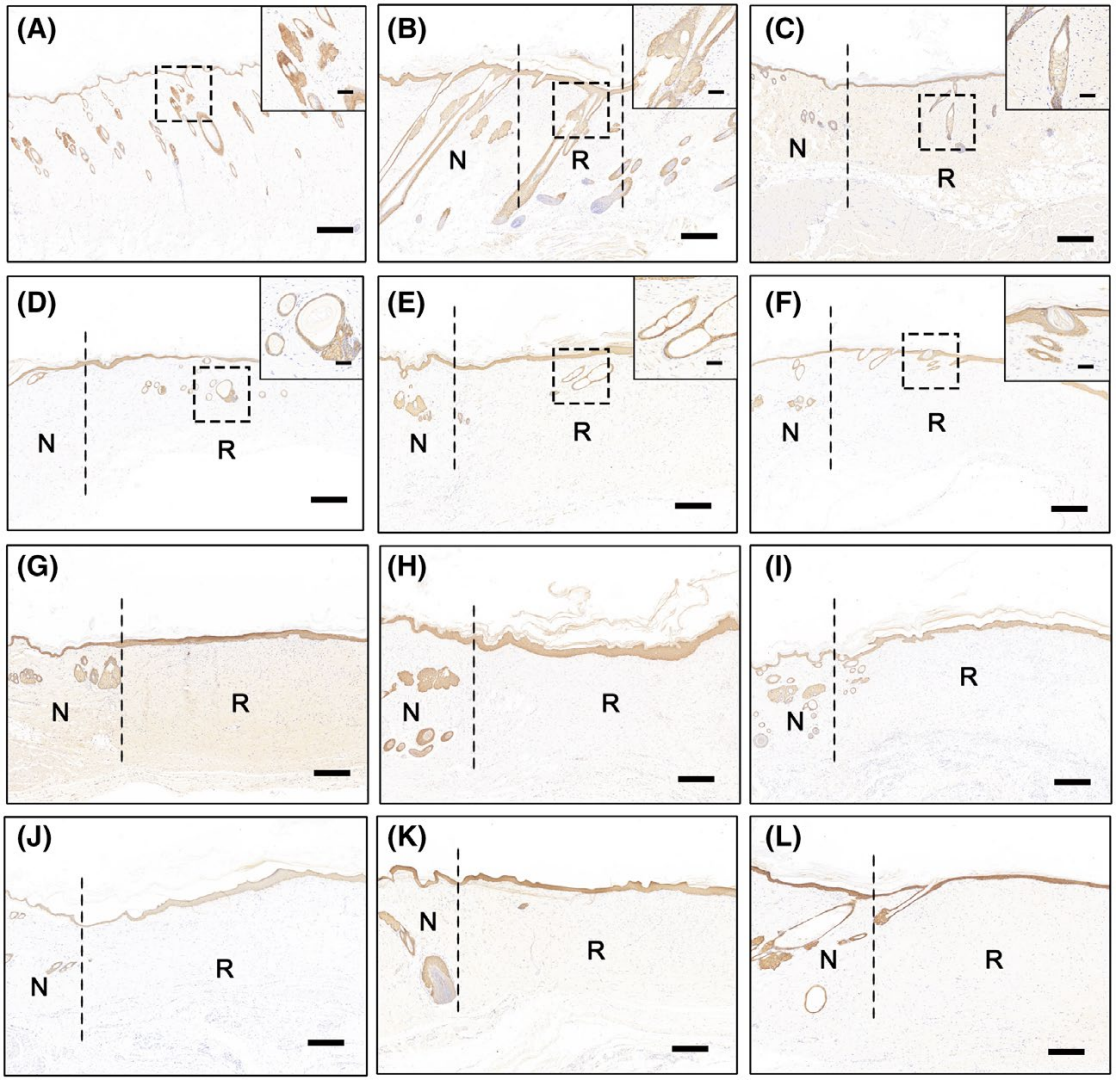
CK14 immunohistochemical staining for the hair follicles and sebaceous glands. (A) represents normal skin, and (B–L) represent the groups with defect diameters of 3–45 mm, respectively. On the left of the black dotted line is normal skin, and on the right is regenerated tissue. N refers to normal tissue. R refers to regenerated tissue. The images were captured at 40× magnification. The scale bar in the lower right corner is 200 μm. The high magnification image in the upper right corner comes from the black dotted box. The images were captured at 200× magnification. The scale bar in the high magnification image of the upper right corner is 50 μm.

CK14 is a marker of the hair follicle and sebaceous gland. To further verify the reconstruction of hair follicles and sebaceous glands, CK14 immunohistochemical staining is performed on the tissue sections. There is CK14‐positive expression in 20% of the central area of the regenerated tissue in the groups with skin defect diameters of 3–15 mm. However, there are no hair follicles and sebaceous gland in 20% of the central area of the regenerated tissue in the groups with defect diameters of 20–45 mm, but scattered hair follicle and sebaceous gland‐like structures are found in the area of regenerated tissues outside the 20% of the central area (Figure 8).

**FIGURE 9:**
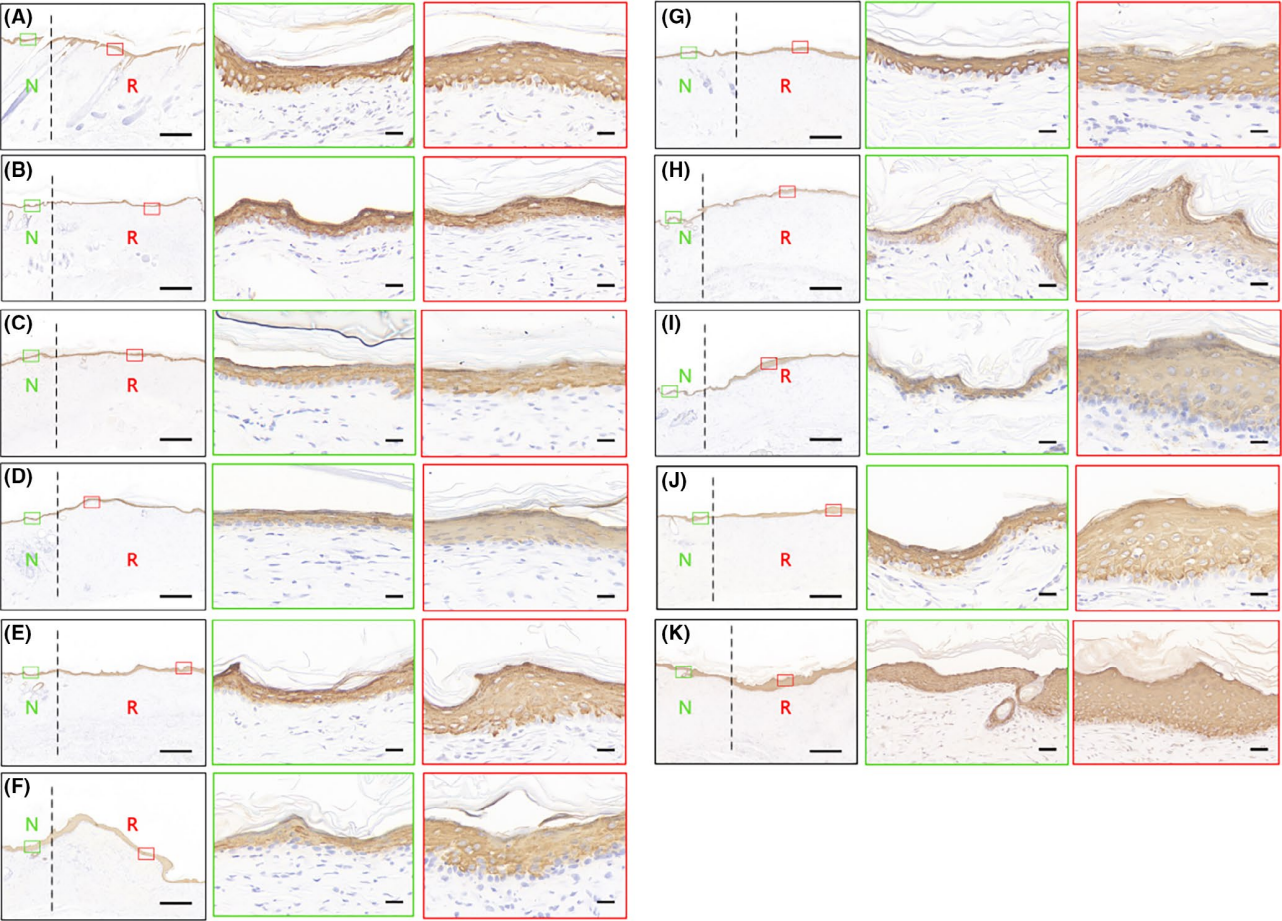
CK10 immunohistochemical staining for epidermal terminal differentiated cells. (A–K) represent the groups with defect diameters of 3–45 mm, respectively. The green box represents the high manification image of normal skin, and the red box represents the high magnificaton image of groups with defect diameters of 3–45 mm, respectively. On the left of the black dotted line is normal skin, and on the right is regenerated tissue. N refers to normal tissue. R refers to regenerated tissue. The scale bar at the bottom right corner of the black box is 200 µm. The images were captured at 200× magnification. Scale bar in the high magnification image in the lower right corner is 50 μm.

CK10 is a marker of epidermal terminal differentiated cells. Epidermal cells differentiating normally play a key role in epidermal normal function. Figure 9 shows that in normal epidermis, CK10 is expressed in the granular cell layer and spinous cell layer and rarely expressed in the basal cell layer. In the regenerated epidermis, CK10 is expressed not only in granular cell layer and spinous cell layer but also in basal layer. It indicates that a large number of epidermal stem cells differentiate into epidermal cells in the basal layer of regenerated epidermis.

**FIGURE 10:**
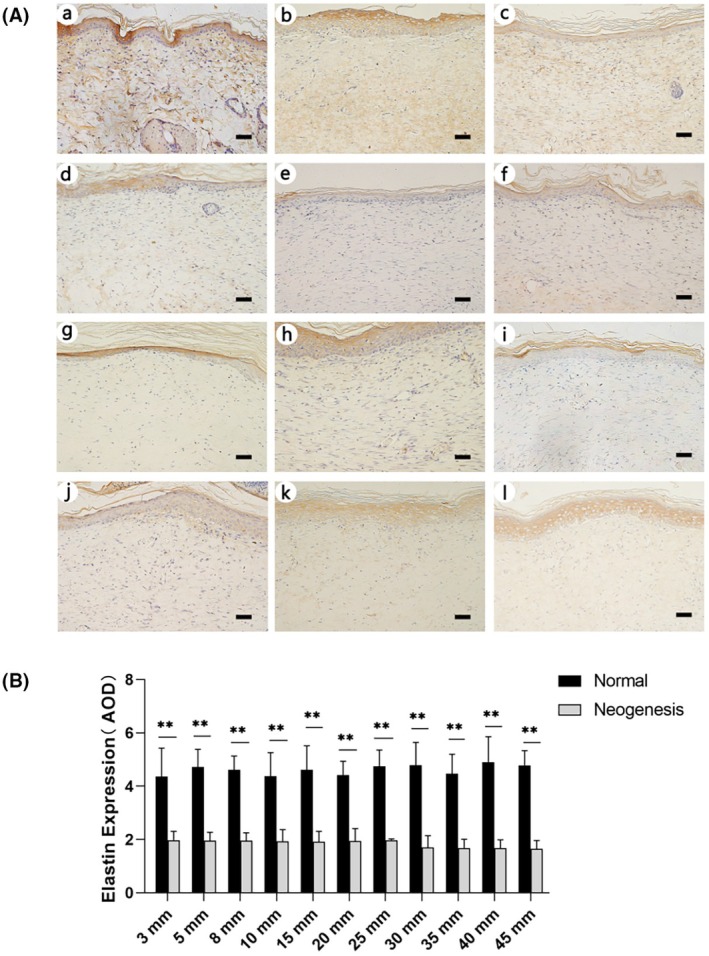
(A) Elastin immunohistochemical staining. (a) represents normal skin, and (b–l) represent the groups with defect diameters of 3–45 mm, respectively. The images were captured at 200× magnification. The scale bar in the lower right corner is 50 μm. (B) Semiquantitative analysis of elastin immunohistochemical staining (**p < 0.01).

Elastin immunohistochemical staining result is shown in Figure 10A. Semiquantitative analysis of elastin DAB staining (Figure 10B) shows that the elastin expression in the groups with skin defect diameters of 3–8 mm is slightly higher than that in the groups with skin defect diameters of 10–45 mm, but there is no significant difference of elastin expression in the regenerated tissue among each group (*p* > 0.05).

### 3.5 Functional repair score for skin injury assessment

**FIGURE 11:**
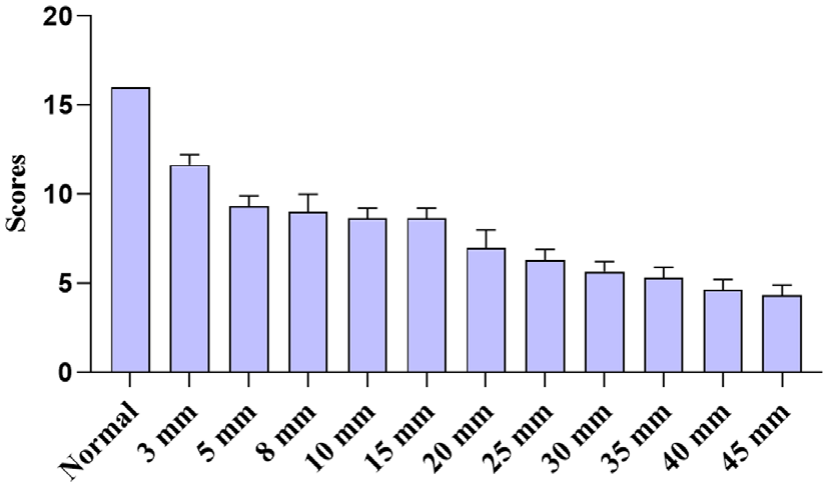
Functional repair scores of rat skin defects.

The functional score result of regenerated skin tissue is shown in Figure 11. The higher the score, the better the skin tissue repair, and the total score for normal skin is 16.

## 4 DISCUSSION

Skin defect size is a key factor in wound healing.^52^ The size of the skin critical defect in the rat model has not been conclusively established. Animal models of skin critical defects are essential for the research of skin repair products and drugs. During wound healing, the body initiates skin repair mechanisms to restore the skin's structural integrity, of which the complete epithelialization of keratinocytes is an important indicator of wound healing.^53^ Although scar healing has some cosmetic and physiological issues, it closes the wound, prevents infection and water loss, achieves the skin's primary barrier function, and maintains its integrity. In this study, we first put forward the concept of skin structure repair based on whether the skin structure restores the integrity of barrier function and define the skin critical defects. We observed that the time of skin structural repair increased with the skin defect diameters. Spontaneous structural repair is achieved in rat groups with skin defect diameters of 3–45 mm. In the group with skin defect diameter of 50 mm, only one rat survived 3 months postoperatively, with a mortality of 90%. This surviving rat underwent repeated scab rupture and loss, fluid exudation, and failed to achieve the structural repair. In this process, the loss of body energy metabolism is greater and the mortality is higher.^54^, ^55^ Therefore, we have primarily defined a skin defect diameter between 45 and 50 mm as a critical value for the spontaneous healing of the rat skin. The exact value of the critical defect in the rat back needs to be further defined within the above range by future experimentation.

The reconstruction of skin accessory structures is very important for the functional recovery of damaged skin.^56^, ^57^, ^58^ The literature has shown that there are almost no hair follicles and sebaceous glands in the scar tissue of adult mammals.^59^ However, our investigation indicates that the regeneration of skin accessory structures is related to the size of defect diameter. In rats with skin defect diameters ≤15 mm, regenerated hair follicles and sebaceous glands were present in the center at 2 weeks after skin structural closure. In the groups with skin defect diameters of 20–45 mm, there is no regenerated hair follicle and sebaceous gland structure in the center of the regenerated tissue, but hair follicle and sebaceous gland‐like structures are present in the regenerated tissue adjacent to normal skin. It can be seen that different wound sizes have crucial impact on the healing outcome.

The repair of skin injury is the restoration of tissue structure and function in different degrees, and there is no systematic quantitative standard yet. With the development of individualized treatment and personalized medicine, the quantification of postoperative effect is the basis for elucidating the significant influence of treatment. At present, skin injury repair is mainly evaluated by the macro and general observation, and the quantitative assessment of micropathology after skin healing is still lacking. Therefore, based on structural repair, we systematically quantified relevant indicators of skin structure according to the regeneration and reconstruction of skin accessory structures, and put forward the quantitative scores of functional repairs for the first time. For the injury of the epidermis and superficial dermis, the repair can be completed by the migration of epidermal cells at the edge of the wound and stem cells in the remaining hair follicles and sweat glands in the dermis.^60^ For full‐thickness skin defects (including epidermis and dermis), normal epidermal cells at the edge of the wound migrate to the center of the wound until the migrating cells meet.^60^, ^61^ Considering the characteristics of epidermal cells migrating to the center, the wound width is divided into five equal parts, and the skin functional repair is quantitatively scored based on the occurrence of skin accessory structures within the above five equal parts. Epidermal epithelialization of the skin is an important step in wound healing.^62^, ^63^ Studies have shown that the thickness of the regenerated epidermis becomes greater after skin injury, and its thickness is negatively correlated with the quality of skin healing.^64^ The skin basement membrane is located between the epidermis and dermis. It closely connects the epidermis and dermis and inhibits the direct contact between the epidermis and dermis. It plays a very important role in maintaining the integrity of the skin. Besides, hair follicles and sebaceous glands play a pivotal role in skin temperature regulation and guiding nerve migration.^56^, ^57^, ^58^ Therefore, the regeneration and reconstruction of the basement membrane, hair follicles, and sebaceous glands, as well as epidermal thickness, are scored by histomorphology, molecular pathology, and computer technology.

Although the quantitative scores of functional repairs are derived from specific studies on rats, in terms of their future extended applications, and may not be directly applicable to mice, rabbits, or large animal models, or even to humans, the underlying concept of quantitatively assessing regenerated accessory structures is expected to be extended to the level of large animals. With the advancement of noninvasive imaging techniques, it will help to visualize the functional reconstruction of human skin tissues, thereby providing a scientific basis for accelerating human skin repair and the research and development of related products.

It should be acknowledged that the quantitative scores of functional repairs established in this study do have certain limitations. Although this scoring system was constructed based on the histological features of the healed wound, it is very likely that subjectivity may be involved when assessing parameters such as the maturity and density of skin appendages, which may lead to differences in the results. In the follow‐up studies, we plan to adopt the double‐blind scoring method and utilize artificial intelligence–assisted image analysis algorithms to minimize the impact of subjective factors on the experimental results as much as possible. In future studies, we will adopt a broader range of research methods, integrating biochemical markers and the evaluation of cellular infiltration in healed tissue into the wound healing assessment criteria.^65^, ^66^ These improvements aim to enable a more accurate and reliable quantitative evaluation. Through refinement of the experimental design, we seek to support further progress in skin repair research.

Of note, histochemistry and immunohistochemistry are the commonly used methods to evaluate the degree of skin healing, but they are all invasive skin pathological diagnosis techniques, which cannot achieve continuous diagnosis of skin healing effect for the same individual. However, the evaluation tool of wound healing should be a systematic method, and a large number of repetitive studies should be performed to obtain the expected quality of the evaluation of functional repair. In the future, it is expected to realize continuous multidimensional evaluation and systematic quantification of skin regeneration and reconstruction using noninvasive skin diagnosis technologies such as three‐dimensional reconstruction of skin surface, high‐resolution ultrasound (US), and skin computed tomography (CT) to further improve the scoring table of skin functional repair.

## 5 CONCLUSIONS

Through the analysis of experimental data, the allowable range of critical skin defect values of SD rats, weighing 180 ± 10 g, is defined as the defect diameter between 45 and 50 mm preliminarily. The concept and category of skin structural and functional repair are put forward; that is, the closure of skin wound, including scar healing, is defined as structural repair, and on this basis, if there is some reconstruction of skin accessory structures, it belongs to the category of functional repair, which makes up for the blank of quantitative evaluation of functional repair. The regression equation between structural skin healing time and defect diameter is clarified and can be used to predict the healing time of skin defects.

These research findings have not only laid the foundation for evaluating the functional repair of skin in rat models but also provided a conceptual paradigm that has the potential to be extended to large animal models. The methods in defining critical‐sized defects and assessing tissue regeneration are expected to accelerate preclinical research related to skin repair and promote the development of skin repair treatment strategies with translational potential.

## AUTHOR CONTRIBUTIONS

**Cong Sun:** Writing – original draft. **Weihong Guo:** Writing – review and editing. **Fang Liang:** Data curation; formal analysis. **Rabia Javed:** Writing – review and editing. **Weijian Hou:** Formal analysis; investigation. **Xingdong Zhang:** Supervision. **Qiang Ao:** Conceptualization; funding acquisition; supervision.

## FUNDING INFORMATION

This work was supported by the National Key Research and Development Program of China (2023YFC2410403).

## CONFLICT OF INTEREST STATEMENT

The authors declare that they have no known competing financial interests or personal relationships that could have appeared to influence the work reported in this paper.

## ETHICS STATEMENT

All animal procedures were approved by the Animal Experiment Ethics Committee of China Medical University (approval number: CMU2019251) and were conducted in accordance with national and institutional guidelines for the care and use of laboratory animals.

## Supporting information


Appendix S1.

## Data Availability

The data that support the findings of this study are available from the corresponding author upon reasonable request.
