# Supplementary material for: Definition of critical skin defect and concepts of structural and functional repairs: Proposal and verification in a rat model
Source: Animal Model Exp Med. 2025 Sep 14;9(1):168–82. doi: 10.1002/ame2.70075 (PMC12907982; doi:10.1002/ame2.70075)
Supplement: Supplementary file 1 — Appendix S1. [file AME2-9-168-s001.docx]

**Supplementary**

1. **Materials and Methods**

## *Animals and experiment groups*

## In this study, 120 healthy male SD rats of SPF grade, weighing 180 ± 10 g, were fed adaptively for 1 week before the operation. The operation was carried out following international standards^1^ and was approved by the animal experiment ethics committee of China Medical University.

The rats were randomly and evenly divided into 12 groups, to prepare circular skin defects with diameters of 3, 5, 8, 10, 15, 20, 25, 30, 35, 40, 45 and 50 mm respectively. The structural repair time of skin wounds with different diameters was observed, and the maximum diameter of skin defect that can structurally close was determined, which was identified as the critical value of skin defect.

## *Gross observation*

The activity, mental state, weight and death of rats was observed every day. The color, secretion and edema of the wound surface were observed and photographed with iPhone 11 (mwne2ch/a) until the scab fell off spontaneously. To ensure the accuracy and standardization of the results, the rats were placed in a fixed position after anesthesia, and the camera angle and distance were consistent each time.

## *Histopathological and morphometric analysis*

Two weeks after skin structural closure, all rats were euthanized by intracardiac injection of 200 mg/kg pentobarbital sodium. The whole layer of the wound healing area including part of the surrounding normal skin was cut off, then the obtained tissue was cut into two pieces along the left and right directions, one piece of the specimens was immersed in 4% paraformaldehyde (Tianjin Guangfu Fine Chemical Research Institute, China) for 24 hours, then embedded in paraffin and cut into slices with a thickness of 5 μm. Hematoxylin and eosin (HE), periodic acid Schiff (PAS), Masson, Elastic Verhoeff-van Gieson (EVG), picrosirius polarization (PSP), and immunohistochemical streptavidin-peroxidase (SP) staining were performed according to the established procedures. The other piece of the specimens was frozen in the refrigerator at - 80 ℃ for oil red O (ORO) staining.

HE staining was used to evaluate the thickness of regenerated epidermis. The left, middle and right visual fields of regenerated epidermis were selected, and Image J software was used to analyze the thickness of the new epidermis. HE staining of the normal skin was used as the control.

## *Immunohistochemical evaluation*

Immunohistochemical streptavidin-peroxidase (SP) was performed using the following agents: CK14 (1:200, rabbit. No. 119695. Abcam) is a marker of the hair follicles and sebaceous glands; CK10 (1:200, ab76318. Abcam) is a marker of epidermal terminal differentiated cells; SP-DAB kit (Fuzhou Maixin Biotechnology Development, China).

The regenerated tissue was divided into five equal parts A, B, C, D and E . The DAB positive expression of brownish yellow or brown in each part was observed and photographed for skin functional repair score. Image-Pro Plus (version 6.0, media cybernetics, USA) was used to determine the area of DAB positive area per unit area in the images for semi-quantitative immunohistochemical analysis.

## *Statistical Analysis*

**General data analysis methods**

All data are presented as Mean ± SD. SPSS software 19.0 was used for statistical analysis. Three or more groups of data were analyzed by one-way analysis of variance (ANOVA). A self-paired t-test was used for self-sample comparison.

**Fitting analysis of the change of** **structural repair time with the skin defect diameter**

**Introduction of fitting analysis methods****.** To explore the relationship between the skin structural repair time and the defect diameter, the size of skin defect diameter and the time of skin structural repair were recorded, and the average days of skin structural repair in different groups with different defect diameters were calculated. The skin defect diameters were defined as independent variables, and the average days of skin structural repair were defined as dependent variables. SPSS software 19.0 was used to draw the scatter diagram for changes of the days of skin structural repair with the skin defects diameters, and curve estimation was used to fit the data, then the optimal regression model was selected and tested.

**Establishment and screening of regression models between skin structural repair time and defect diameters.** SPSS software 19.0 was used to calculate the judgment coefficient R^2^ of different regression models, and regression models were selected preliminarily based on coefficient R^2^.

The selection indexes of the optimal regression model include Root Mean Square Error (RMSE), Accuracy factor (A_f_) and Bias factor (B_f_). RMSE is a numerical indicator that describes the dispersion degree of the predicted value of the regression model. It quantifies the overall difference between the observed value and the predicted value fitted by the regression model. The smaller the difference, the smaller the dispersion degree of the predicted value of the regression model and the higher the fitting degree of the regression model. A_f_ represents the average accuracy of the predicted value relative to the observed value. B_f_ represents the average deviation of the predicted value from the observed value. The closer the value of A_f_ and B_f_ are to 1, the closer the predicted value is to the observed value and the higher is the fitting degree of the regression model. The predicted value and observed value of skin structural repair time were put into the following formula to calculate RMSE, A_f_ and B_f_ .^2,3^


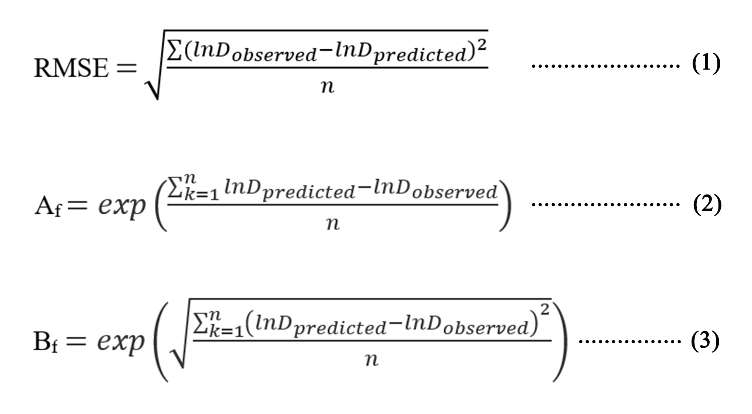


In the above formula, *n* represents the total number of animals, *D_predicted_* represents the predicted time of skin structural repair by the regression models (days), and *D_observed_* represents the observed time of skin structural repair (days).

**Validation and analysis of the optimal regression model between skin structural repair time and defect diameters****.** Through the optimal regression model, the prediction time of skin structural repair under different defect diameters is calculated, then the Relative error and Absolute error between the predicted value and observed value are calculated.

To verify the rationality and feasibility of the optimal regression model, we add three groups of animal models with skin defect diameter of 13 mm, which was not included in the above fitting sample diameters. Then the time required for skin structural repair was recorded, and the relative error and absolute error were calculated through the optimal regression equation. The three groups of animal models are as the following: (1) control group (spontaneous healing), (2) matrigel group (matrigel covers defect), and (3) AEC group (Autologous Epithelial Cells + matrigel).

Isolation of rat Autologous Epithelial Cells. The SD rats are anesthetized, shaved, and the dorsal of the rats is disinfected. A skin sample of approximately 13 mm in diameter is excised from the back, placed in pre-prepared PBS containing 1% double antibodies, and immediately process in the clean bench. The skin mucosa and blood vessels are removed, and the remaining skin is cut into 1 mm-wide strips. The strips are washed three times with PBS containing double antibodies, and then placed in a 0.2% neutral protease solution for 2 hours at 37°C. The epidermis and dermis are then separated, and the epidermis is cut into small pieces and placed in a 0.25% trypsin solution for 8 minutes at 37°C. The reaction is stopped with serum, and the epidermal tissue is removed using a cell filter. The remaining solution is centrifuged at 1000 rpm and 4°C for 5 minutes. The cells are resuspended with KM culture medium and transferred to a cell culture flask. After three days, cell adhesion could be observed.

The activity of autologous epidermal cells is assessed through Acridine orange and Propidium iodide (AO/PI) staining assay. AO/PI Staining Apoptosis Detection Kit (BB-4142-1, Bestbio, Shanghai, China, <http://www.bestbio.com.cn/)> The basic steps are as follows: (1) prepare AO and PI staining solutions. Reagent C (100 μL) is mixed with sterile deionized water (900 μL) to make staining binding buffffer. Cells (1 × 10^6^ ) are washed twice with PBS and resuspended in 500 μL staining binding buffffer; (2) AO staining solution (5 μL) and PI staining solution (5 μL) are added to cells in turn and mixed gently, following which the epidermal cells (1000 r/min, 5 min) cells are incubated at 4°C in a dark room for 15 min. (3) Epidermal cells cells are then rinsed by PBS and observed under a fluorescence microscope.

The rat dorsal wound of control group is normally adopted without any treatment; Matrigel group is administered 400 μL matrigel on the wound bed; AEC group, 200 μL matrigel is inoculated with 200 μL cell suspension with cell density of 2 × 10^6^/mL, and each rat is injected on the wound bed with 400 μL into the wound with cell density of 1 × 10^6^/mL.

# Results


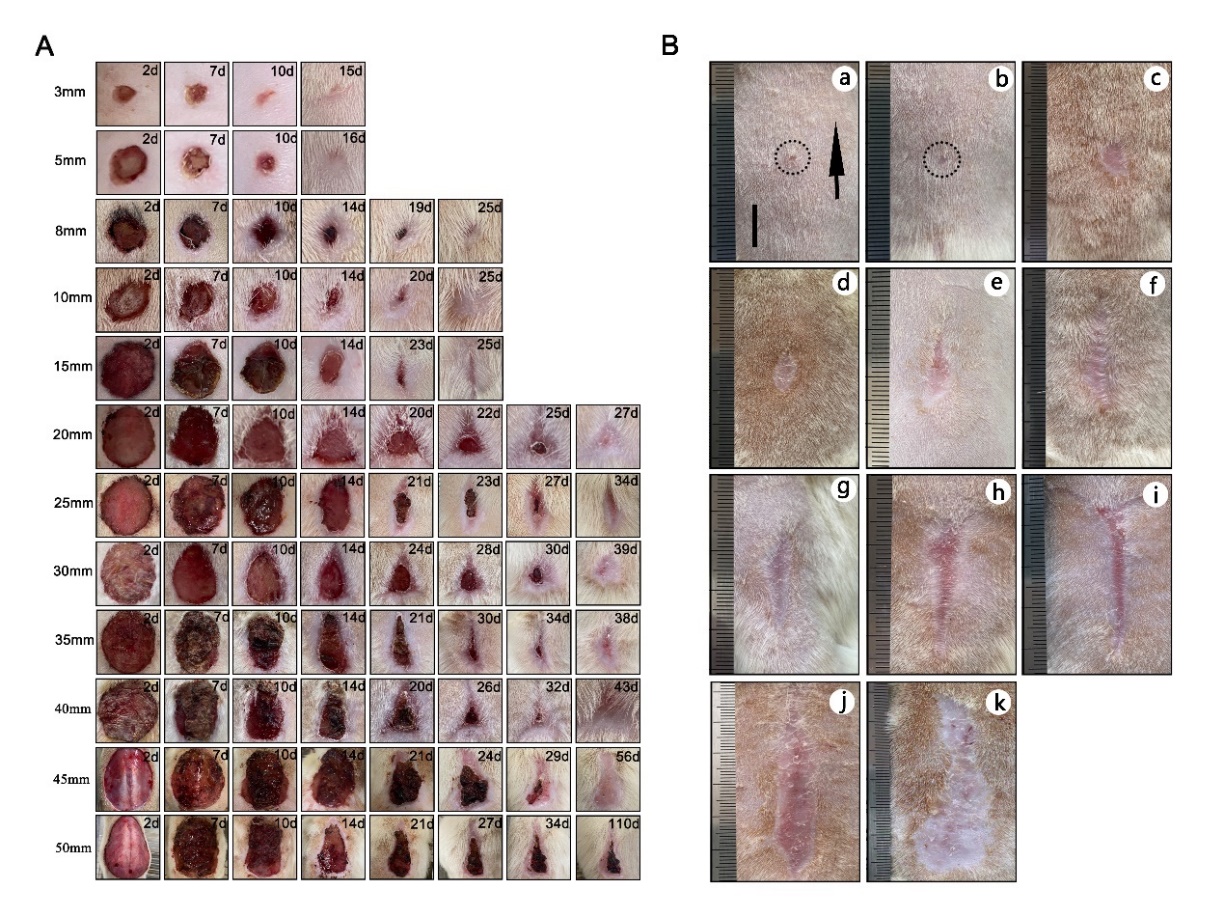


**Figure S1**

1. **Representative photos of the skin structural repair with different skin defect diameters.** In the groups with skin defect diameters of 3-45 mm, the epidermal epithelialization has completed and the structural closure of the wound has been analyzed. In the group with defect diameter of 50 mm, the structural closure of the wound is not completed in 110 days.
2. **Representative general appearance of the wound after structural repair.** In the image, a-k represent the groups with skin defect diameters of 3, 5, 8, 10, 15, 20, 25, 30, 35, 40 and 45 mm respectively. The inside of the black dotted circle is the appearance of the healed tissue scar of the skin defect diameters 3 mm and 5 mm group. Scale bar in the lower left corner of the image is 10 mm.

**1.2 Fitting results of skin structural repair time and defect diameters**

*Establishment and screening of regression models between skin structural repair time and defect diameters.* curve estimation is used to obtain 11 regression models (Table S1).

**Table S1 Main statistical data and ANOVA test results of 11 models**

| **Models** | **Model summaries** | | |
| --- | --- | --- | --- |
|  | **R^2^** | **F** | **Sig.** |
| Cubic | .955 | 496.317 | .000 |
| Quadratic | .933 | 494.819 | .000 |
| Linear | .921 | 834.388 | .000 |
| Compound | .912 | 742.747 | .000 |
| Growth | .912 | 742.747 | .000 |
| Exponential | .912 | 742.747 | .000 |
| Logarithmic | .912 | 742.747 | .000 |
| Power | .903 | 671.094 | .000 |
| Logistic | .799 | 285.946 | .000 |
| S | .715 | 180.993 | .000 |
| Inverse | .554 | 89.460 | .000 |

**References**

1. Cima G. AVMA Guidelines for the Euthanasia of Animal: 2013 Edition. Javma-J Am Vet Med A. 2013; 242: 715-6.
2. Baranyi J, [Pin](https://pubmed.ncbi.nlm.nih.gov/?term=Pin+C&cauthor_id=10443535) C, Ross, T. Validating and comparing predictive models. Int J Food Microbiol. 1999; 48: 159-66.
3. Huertas JP, Ros-Chumillas M, Garre A, Fernandez PS, Aznar A, Iguaz A, et al. Impact of Heating Rates on Alicyclobacillus acidoterrestris Heat Resistance under Non-Isothermal Treatments and Use of Mathematical Modelling to Optimize Orange Juice Processing. Foods. 2021; 10.
